# Supplementary figures and images for: Evolutionary radiation strategy revealed in the Scarabaeidae with evidence of continuous spatiotemporal morphology and phylogenesis
Source: Commun Biol. 2024 Jun 5;7:690. doi: 10.1038/s42003-024-06250-1 (PMC11153540; doi:10.1038/s42003-024-06250-1)

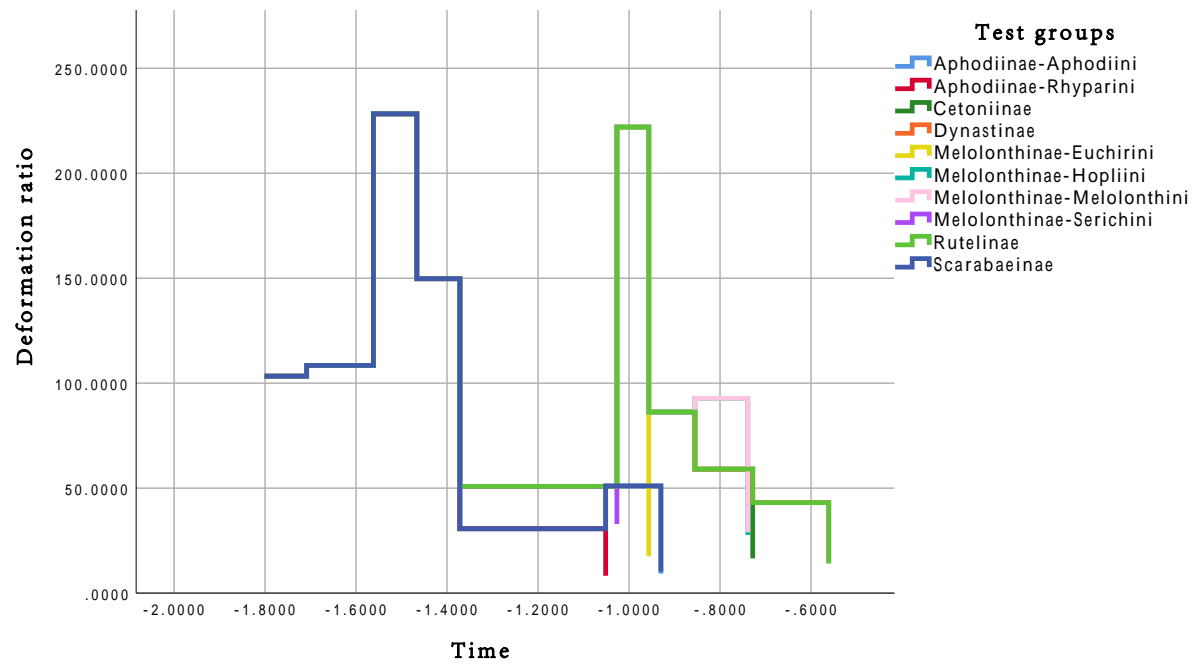

Supplement: Supplementary file 2 — Supplementary Data 1 [file 42003_2024_6250_MOESM2_ESM.zip › Supplementary Data 1/No.2 DR-Mandible test-Euclidean distance.pdf]

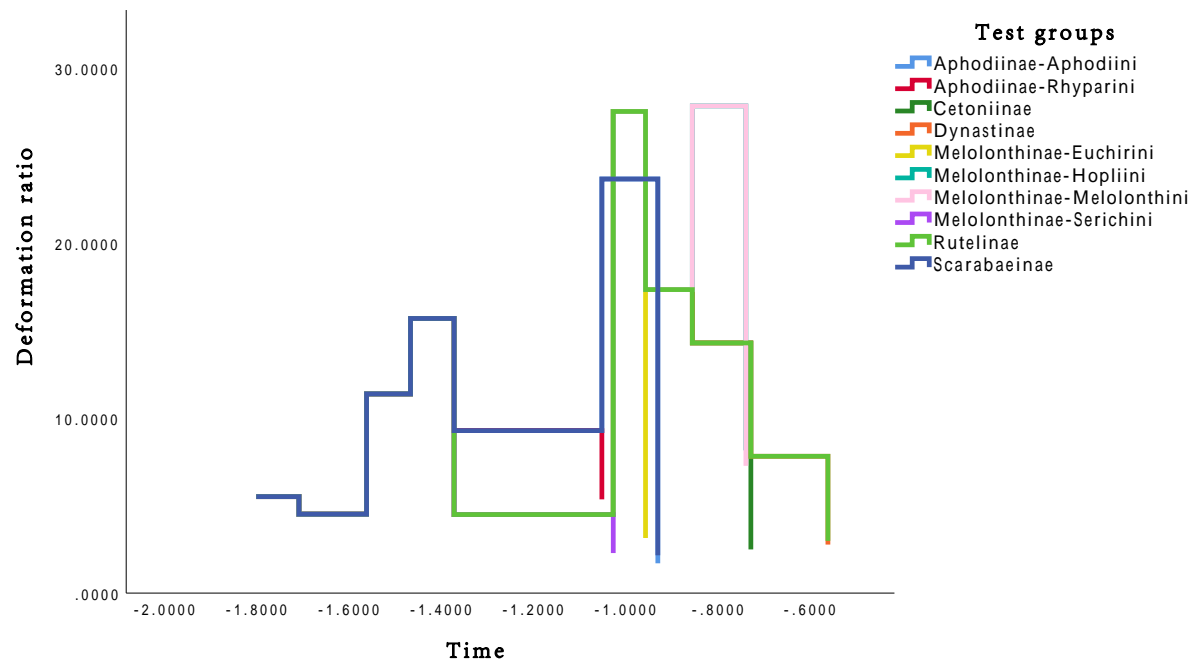

Supplement: Supplementary file 2 — Supplementary Data 1 [file 42003_2024_6250_MOESM2_ESM.zip › Supplementary Data 1/No.3 DR-Pronotum test-Mahalanobis distance.pdf]

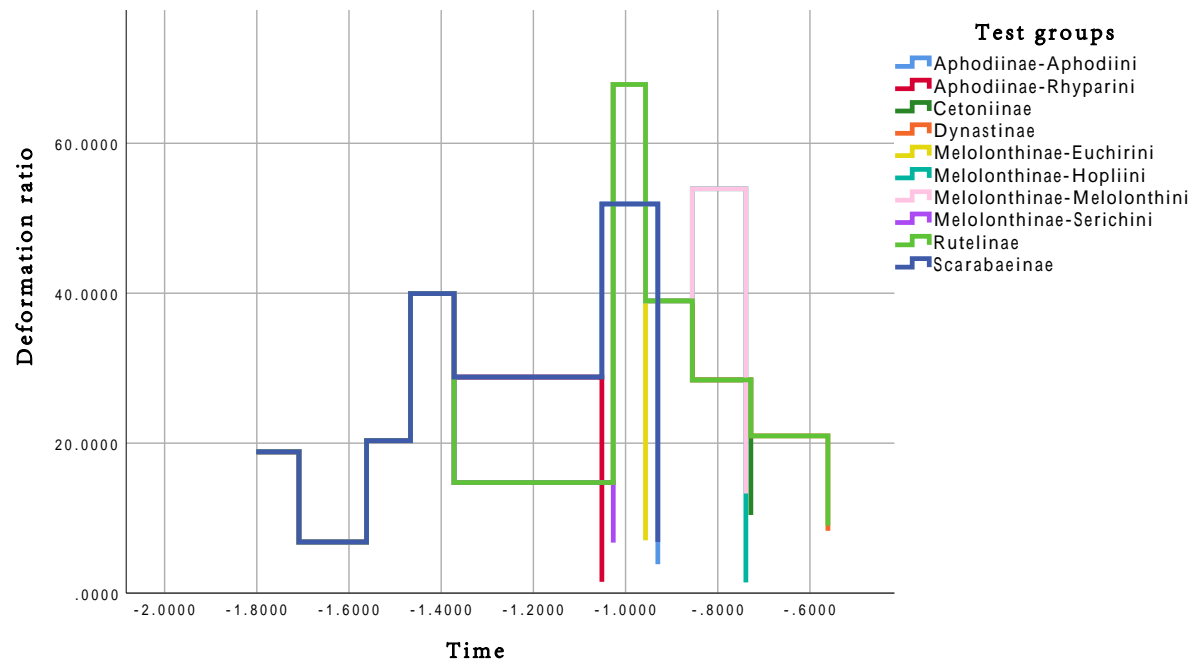

Supplement: Supplementary file 2 — Supplementary Data 1 [file 42003_2024_6250_MOESM2_ESM.zip › Supplementary Data 1/No.4 DR-Pronotum test-Euclidean distance.pdf]

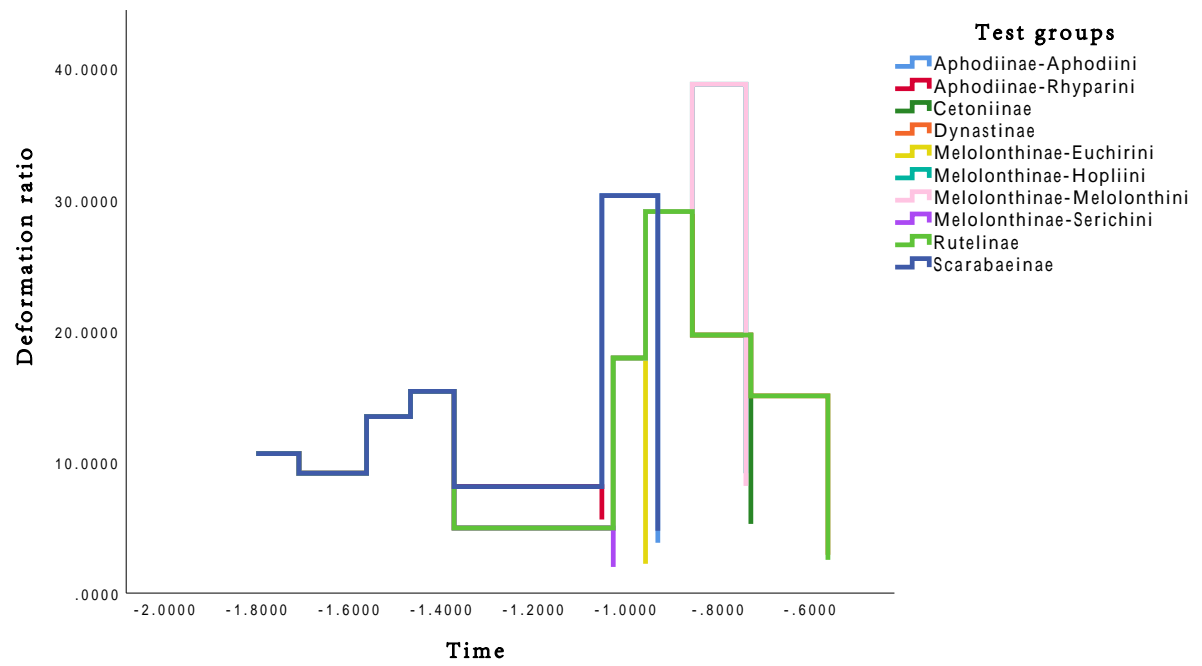

Supplement: Supplementary file 2 — Supplementary Data 1 [file 42003_2024_6250_MOESM2_ESM.zip › Supplementary Data 1/No.5 DR-Elytron test-Mahalanobis distance.pdf]

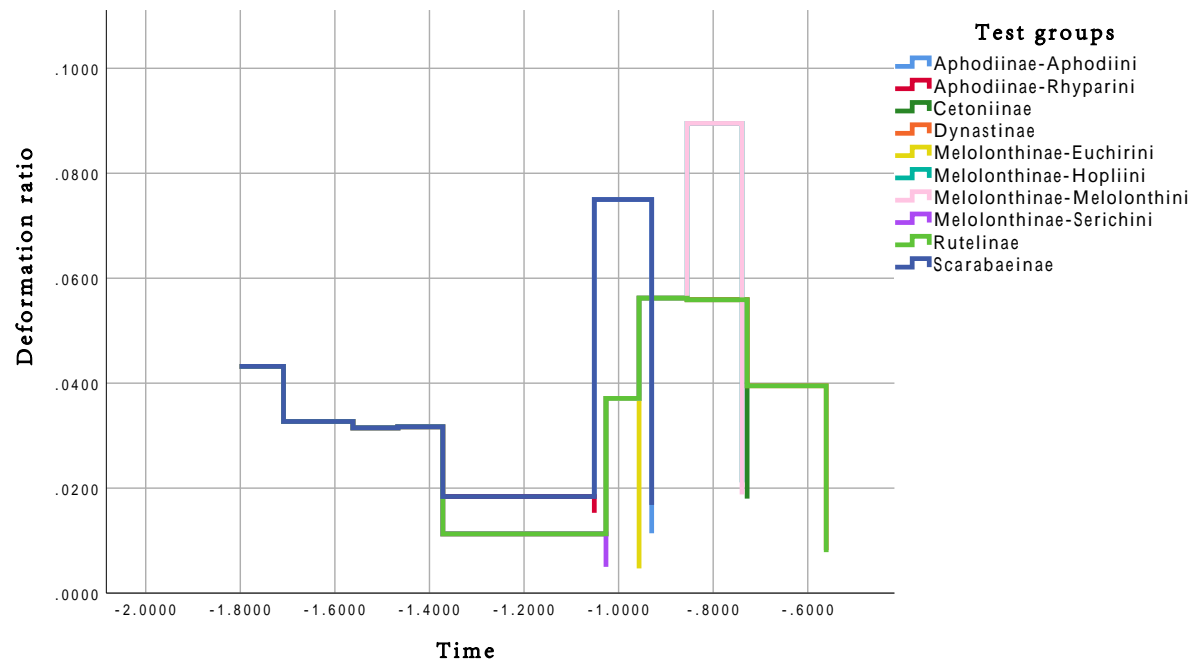

Supplement: Supplementary file 2 — Supplementary Data 1 [file 42003_2024_6250_MOESM2_ESM.zip › Supplementary Data 1/No.6 DR-Elytron test-Euclidean distance.pdf]

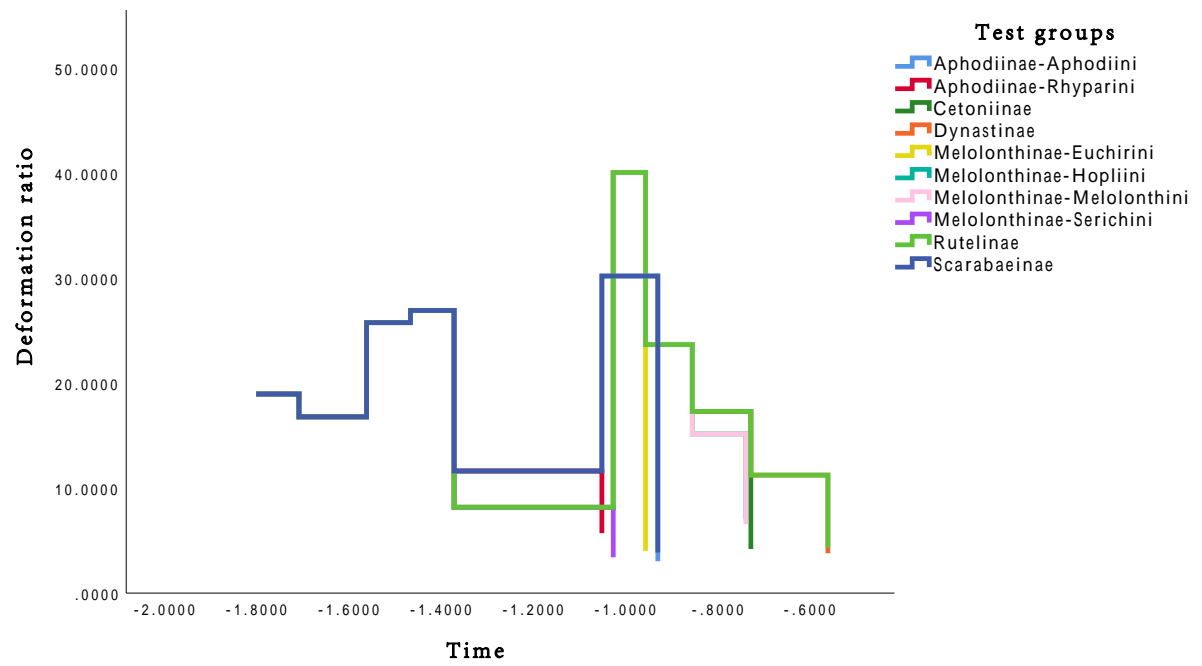

Supplement: Supplementary file 2 — Supplementary Data 1 [file 42003_2024_6250_MOESM2_ESM.zip › Supplementary Data 1/No.7 DR-Hindwing test-Mahalanobis distance.pdf]

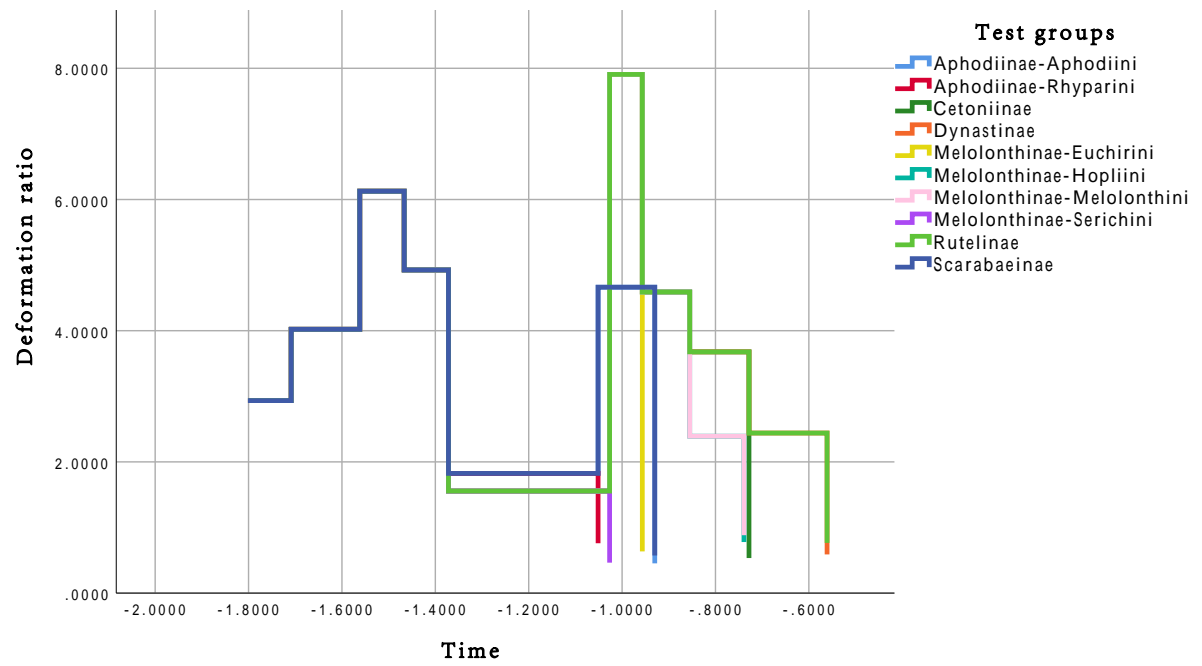

Supplement: Supplementary file 2 — Supplementary Data 1 [file 42003_2024_6250_MOESM2_ESM.zip › Supplementary Data 1/No.8 DR-Hindwing test-Euclidean distance.pdf]
