# Supplementary material for: Evolutionary radiation strategy revealed in the Scarabaeidae with evidence of continuous spatiotemporal morphology and phylogenesis
Source: Commun Biol. 2024 Jun 5;7:690. doi: 10.1038/s42003-024-06250-1 (PMC11153540; doi:10.1038/s42003-024-06250-1)
Supplement: Supplementary file 2 — Supplementary Data 1 [file No.1 DR-Mandible test-Mahalanobis distance.pdf › Supplementary Data 1/No.1 DR-Mandible test-Mahalanobis distance.pdf]

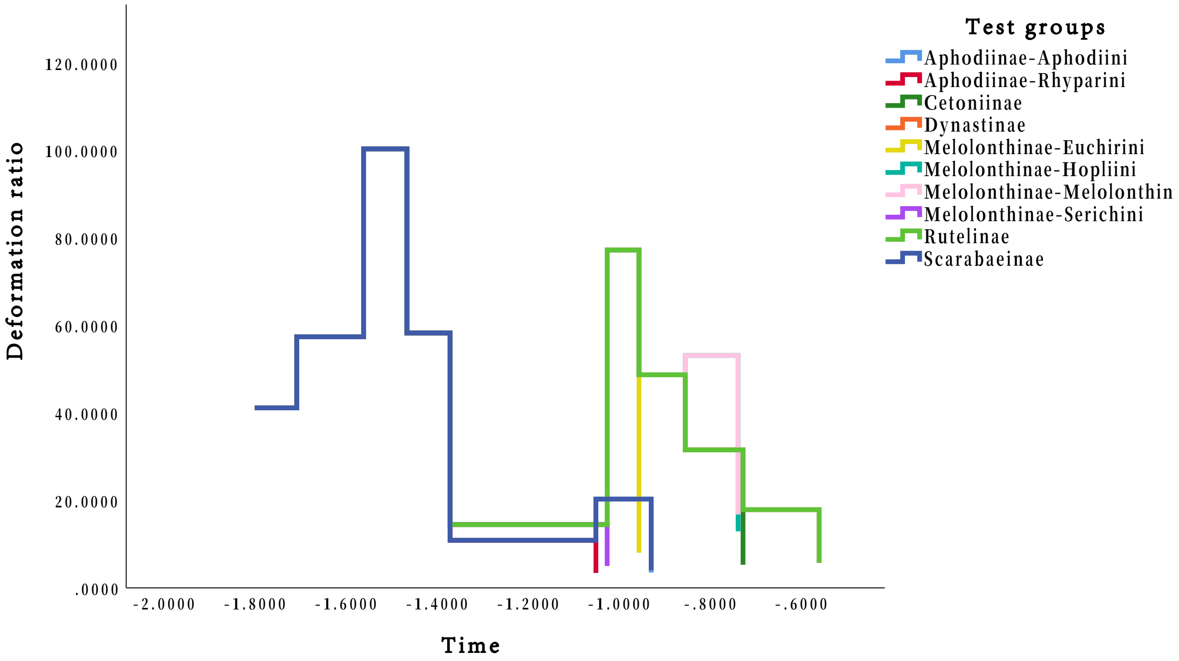

Deformation ratio
120.0000
100.0000
80.0000
60.0000
40.0000
20.0000
.0000
-2.0000
-1.8000
-1.6000
-1.4000
-1.2000
-1.0000
-.8000
-.6000
Time
Test groups
Aphodiinae-Aphodiini
Aphodiinae-Rhyparini
Cetoniinae
Dynastinae
Melolonthinae-Euchirini
Melolonthinae-Hopliini
Melolonthinae-Melolonthin
Melolonthinae-Serichini
Rutelinae
Scarabaeinae
